# Synthesis, structure, and photovoltaic property of a nanocrystalline 2H perovskite-type novel sensitizer (CH_3_CH_2_NH_3_)PbI_3_

**DOI:** 10.1186/1556-276X-7-353

**Published:** 2012-06-28

**Authors:** Jeong-Hyeok Im, Jaehoon Chung, Seung-Joo Kim, Nam-Gyu Park

**Affiliations:** 1 School of Chemical Engineering and Department of Energy Science, Sungkyunkwan University, Suwon, 440-746, Republic of Korea; 2 Department of Chemistry, Division of Energy Systems Research, Ajou University, Suwon, 443-749, Republic of Korea

**Keywords:** (CH_3_CH_2_NH_3_)PbI_3_, 2H perovskite, Dye-sensitized solar cell, Nanodot, Sensitizer

## Abstract

A new nanocrystalline sensitizer with the chemical formula (CH_3_CH_2_NH_3_)PbI_3_ is synthesized by reacting ethylammonium iodide with lead iodide, and its crystal structure and photovoltaic property are investigated. X-ray diffraction analysis confirms orthorhombic crystal phase with *a* = 8.7419(2) Å, *b* = 8.14745(10) Å, and *c* = 30.3096(6) Å, which can be described as 2 H perovskite structure. Ultraviolet photoelectron spectroscopy and UV-visible spectroscopy determine the valence band position at 5.6 eV versus vacuum and the optical bandgap of *ca.* 2.2 eV. A spin coating of the CH_3_CH_2_NH_3_I and PbI_2_ mixed solution on a TiO_2_ film yields *ca.* 1.8-nm-diameter (CH_3_CH_2_NH_3_)PbI_3_ dots on the TiO_2_ surface. The (CH_3_CH_2_NH_3_)PbI_3_-sensitized solar cell with iodide-based redox electrolyte demonstrates the conversion efficiency of 2.4% under AM 1.5 G one sun (100 mW/cm^2^) illumination.

## Background

Semiconductor nanocrystals have received much attention due to quantum confinement effect, in which the continuous optical transitions between the electronic bands in the bulk crystals become discrete in the nanocrystals and thereby the properties of the nano-sized materials become size-dependent [1-3]. The size-dependent optical properties of semiconductor n anoparticles have been widely applied in displays [4], biomedical imaging sensors [5], and photovoltaic solar cells [6]. In the case of solar cell application, semiconductor nanomaterials can be used as a light-absorbing material (photosensitizer) in either a solid-state pn junction structure or a photoelectrochemical junction type [7]. Dispersion of semiconductor nanocrystal on a high-surface-area n-type or p-type support is an effective method to utilize it as a photosensitizer. For this reason, semiconductor (or quantum dot)-sensitized solar cell has recently attracted a lot of interest [8,9]. As photosensitizers in the semiconductor-sensitized solar cell, metal chalcogenides have been mostly studied, where Sb_2_S_3_-sensitized solar cell demonstrated a conversion efficiency as high as 6.18% at simulated one sun (100 mW/cm^2^) illumination [10]. Recently, a conversion efficiency of 6.54% at one sun was reported based on perovskite semiconductor (CH_3_NH_3_)PbI_3_[11], where (CH_3_NH_3_)PbI_3_ was found to form *in situ* on a nanocrystalline TiO_2_ surface from spin coating of the CH_3_NH_3_I and PbI_2_ mixed solution. Moreover, an organic–inorganic hybrid perovskite structure has advantage over other crystal structures as for the sensitizer since it has high light absorption property and thermal stability as well. Since the perovskite ABX_3_ structure was known to be stabilized depending on the ionic radii of A and B cations in relation with tolerance factor [12,13], it can be possible to tailor a new perovskite-type semiconductor sensitizer by substituting methylammonium cation in the cuboctahedral A site with longer alkyl-chain ammonium cations. Change in the A-site cation is expected to tune the bandgap energy of alkylammonium lead iodide perovskite sensitizer due to change in chemical boding nature. Here, we report for the first time on the synthesis and structural analysis of (CH_3_CH_2_NH_3_)PbI_3_. Valence band position and optical bandgap are evaluated by ultraviolet photoelectron spectroscopy (UPS) and UV-visible (UV–vis) spectroscopy, respectively. Photovoltaic performance of a (CH_3_CH_2_NH_3_)PbI_3_-sensitized solar cell is investigated in the presence of an iodide-based redox electrolyte.

## Methods

The semiconductor sensitizer of (CH_3_CH_2_NH_3_)PbI_3_ was prepared by direct deposition of the γ-butyrolactone (Aldrich, Sigma-Aldrich Corporation, St. Louis, MO, USA) solution with equimolar CH_3_CH_2_NH_3_I and PbI_2_ on a nanocrystalline TiO_2_ surface. CH_3_CH_2_NH_3_I was synthesized by reacting 18.2 mL of ethylamine (2.0 M in methanol, Aldrich) and 10 mL of hydroiodic acid (57 wt.% in water, Aldrich) in a 250-mL round-bottomed flask at 0°C for 2 h. The precipitate was collected by evaporation at 80°C for 1 h, which is followed by washing three times with diethyl ether and then finally dried at 100°C in a vacuum oven for 24 h. The synthesized CH_3_CH_2_NH_3_I powder was mixed with PbI_2_ (Aldrich) at a 1:1 mole ratio in γ-butyrolactone at 80°C for 2 h, which was used as a coating solution for the *in situ* formation of (CH_3_CH_2_NH_3_)PbI_3_ on the TiO_2_ surface. The concentration of the coating solution was 42.17 wt.%, which contains 2.234 g of CH_3_CH_2_NH_3_I (12.9 mmol) and 6.016 g of PbI_2_ (12.9 mmol) in 10 mL of γ-butyrolactone.

Nanocrystalline TiO_2_ particles were prepared by hydrothermal method at 230°C, and non-aqueous TiO_2_ paste was prepared according to the method reported elsewhere [14]. Fluorine-doped tin oxide (FTO) conductive glass (TEC-8, 8 Ω/sq, Pilkington, St Helens, UK) was pre-treated with 0.1 M Ti(IV) bis(ethyl acetoacetato)-diisopropoxide (Aldrich) in 1-butanol (Aldrich) solution, in which the nanocrystalline TiO_2_ paste was deposited and heated at 550°C for 1 h. The thicknesses of the annealed TiO_2_ films were determined by an alpha-step IQ surface profiler (KLA-Tencor Corporation, Milpitas, CA, USA). The perovskite coating solution was spread on the annealed TiO_2_ film (38.46 μL/cm^2^) and was spun for 10 s at a speed of 2,000 rpm in ambient atmosphere. The perovskite (CH_3_CH_2_NH_3_)PbI_3_ formed on the TiO_2_ surface was dried at 100°C for 15 min. Pt counter electrode was prepared by spreading a droplet of 7 mM H_2_PtCl_6_*x*H_2_O in 2-propanol on a FTO substrate and heated at 400°C for 20 min in air. The (CH_3_CH_2_NH_3_)PbI_3_-sensitized TiO_2_ working electrode and the counter electrode were sandwiched using 25-μm-thick Surlyn (SX1170-25, Solaronix SA, Aubonne, Switzerland). The redox electrolyte was prepared by dissolving 0.9 M LiI (Aldrich), 0.45 M I_2_ (Aldrich), 0.5 M *tert*-butylpyridine(Aldrich), and 0.05 M urea (Aldrich) in ethyl acetate (Aldrich), which was introduced into the space of the sealed electrodes prior to measurement.

Powder X-ray diffraction (XRD) profiles were recorded on a Rigaku D/MAX-2200/PC diffractometer (Tokyo, Japan) using graphite-monochromated CuKα radiation (*λ* = 1.5418 Å). Data were collected over the 2*θ* range from 5° to 100° for 4 s in each 0.02° step at ambient temperature. The TREOR software [15] was used for indexing and determining the lattice parameters. For XRD measurement, (CH_3_CH_2_NH_3_)PbI_3_ powder was obtained by drying the solution of the equimolar mixture of CH_3_CH_2_NH_3_I and PbI_2_ at 100°C. Photocurrent and voltage were measured from a solar simulator equipped with a 450-W xenon lamp (6279NS, Newport Corporation, Irvine, CA, USA) and a Keithley 2400 source meter (Cleveland, OH, USA). Light intensity was adjusted with the NREL-calibrated Si solar cell having KG-2 filter for approximating one-sun light intensity (100 mW/cm^2^). While measuring current and voltage, the cell was covered with a black mask having an aperture, where the aperture area was slightly smaller than the active area. Distribution of perovskite (CH_3_CH_2_NH_3_)PbI_3_ in the TiO_2_ film was investigated by a distribution mapping technique using an energy-dispersive X-ray spectroscope (EDS) combined with a field-emission scanning electron microscope (FE-SEM, Jeol JSM 6700 F). X-ray energies corresponding to Ti, Pb, and I were collected as the SEM scanned the electron beam over the surface and cross-sectional area in the TiO_2_ film. The X-ray data were synchronized with the SEM image, and an elemental mapping was created showing the presence of the selected element throughout the selected area. Transmission electron microscope (TEM) image was investigated using high-resolution TEM (HR-TEM, Jeol, JEM-2100 F) at an acceleration voltage of 200 kV. The UV–vis reflectance spectra of the powdered (CH_3_CH_2_NH_3_)PbI_3_, the (CH_3_CH_2_NH_3_)PbI_3_-adsorbed TiO_2_ nanoparticle, and the bare TiO_2_ particle were recorded using a UV/VIS/NIR spectrophotometer (Lambda 950 model, PerkinElmer, Waltham, MA, USA) in a wavelength of 200 to 1,100 nm. UPS equipped with He-I source (hν = 21.22 eV) (AXIS Nova, Kratos Analytical Ltd., Manchester, UK) was used to determine the valence band energy of (CH_3_CH_2_NH_3_)PbI_3_.

## Results and discussion

**Figure 1:**
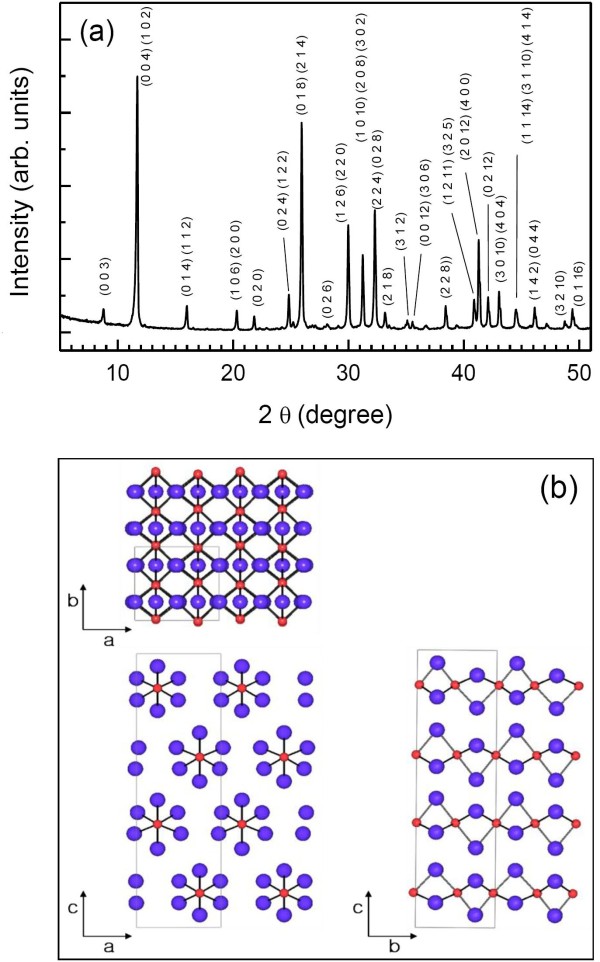
**Powder XRD pattern (a) and crystal structure (b) of (CH**_**3**_**CH**_**2**_**NH**_**3**_**)PbI**_**3**_**.** Red and purple spheres represent Pb and I ions, respectively.

Figure 1a shows the XRD pattern of the synthesized (CH_3_CH_2_NH_3_)PbI_3_. All reflections are indexed by an orthorhombic unit cell with *a* = 8.7419(2) Å, *b* = 8.14745(10) Å, *c* = 30.3096(6) Å. Reflection conditions (*h* + *k* = 2*n* for *hk*0, *h* = 2*n* for *h*00, and *k* = 2*n* for 0*k*0) observed in the XRD pattern indicate that possible space groups are P2_1_mn and Pmmn (Table 1). By assuming a centrosymmetric space group Pmmn, the structural parameters for heavy atoms such as Pb and I are determined by applying the direct method using the EXPO software [16] and refined by the Rietveld method with the FULLPROF program [17]. Table 2 shows the atomic coordinates, isotropic temperature factors, and agreement factors. The structural information about C, N, and H atoms could not be obtained due to the low resolution of the laboratory XRD equipment. As shown in Figure 1b, the structure of (CH_3_CH_2_NH_3_)PbI_3_ can be described as a 2 H perovskite type which consists of infinite chains of face-sharing (PbI_6_) octahedra running along the *b*-axis of the unit cell. These chains are separated from one another by ethylammonium ions.

**Table 1:** **Miller indices (*hkl*), spacing of lattice plane (*d*), and XRD peak intensity (*I*) of (CH**_**3**_**CH**_**2**_**NH**_**3**_**)PbI**_**3**_

***hkl***	***d***_**obs**_	***d***_**cal**_	***I***_**obs**_
(0 0 3)	10.06	10.08	9
(0 0 4) (1 0 2)	7.552	7.552	100
(0 1 4) (1 1 2)	5.532	5.530	11
(1 0 6) (2 0 0)	4.357	4.360	9
(0 2 0)	4.057	4.060	6
(0 2 4) (1 2 2)	3.574	3.576	15
(0 1 8) (2 1 4)	3.422	3.424	82
(0 2 6)	3.158	3.161	3
(1 2 6) (2 2 0)	2.97	2.972	43
(1 0 10) (2 0 8) (3 0 2)	2.852	2.855	30
(0 2 8) (2 2 4)	2.763	2.765	48
(2 1 8)	2.691	2.693	8
(3 1 2)	2.548	2.693	4
(0 0 12) (3 0 6)	2.516	2.518	4
(2 2 8)	2.334	2.335	10
(1 2 11) (3 2 5)	2.199	2.201	12
(2 0 12) (4 0 0)	2.179	2.180	34
(0 2 12)	2.139	2.140	13
(3 0 10) (4 0 4)	2.093	2.095	15
(1 1 14) (3 1 10) (4 1 4)	2.028	2.028	9
(0 4 4) (1 4 2)	1.961	1.961	10
(3 2 10)	1.863	1.862	5
(0 1 16)	1.838	1.846	9

**Table 2:** **Unit cell, positional, and thermal parameters for (CH**_**3**_**CH**_**2**_**NH**_**3**_**)PbI**_**3**_

**Space group: Pmmn**					
***a*** **= 8.7419(2) Å,*b*** **= 8.14745(10) Å,*c*** **= 30.3096(6) Å,*Z*** **= 8**					
***R***_**p**_ **= 15.3%*R***_**wp**_ **= 21.0%*R***_**exp**_ **= 9.47%*χ***^**2**^ **= 4.9**					
	**Site**	***x***	***y***	***z***	***B*(Å**^**2**^**)**^**a**^
Pb1	4*e*	0.75	0.5261(17)	0.1256(12)	0.66(15)
Pb2	4*e*	0.25	0.493(2)	0.3776(10)	0.66(15)
I1	2*a*	0.25	0.25	0.466(2)	3.4(2)
I2	2*a*	0.75	0.75	0.046(2)	3.4(2)
I3	2*b*	0.75	0.25	0.208(2)	3.4(2)
I4	2*b*	0.25	0.75	0.288(2)	3.4(2)
I5	4*f*	0.997(6)	0.25	0.0898(13)	3.4(2)
I6	4*f*	0.993(6)	0.75	0.1693(13)	3.4(2)
I7	4*f*	0.526(6)	0.25	0.3429(12)	3.4(2)
I8	4*f*	0.013(6)	0.75	0.4195(14)	3.4(2)

Structural parameters for C, N, and H atoms were not refined. ^a^All of the isotropic atomic displacement parameters (*B*) of each atomic species were constrained to have the same values.

**Figure 2:**
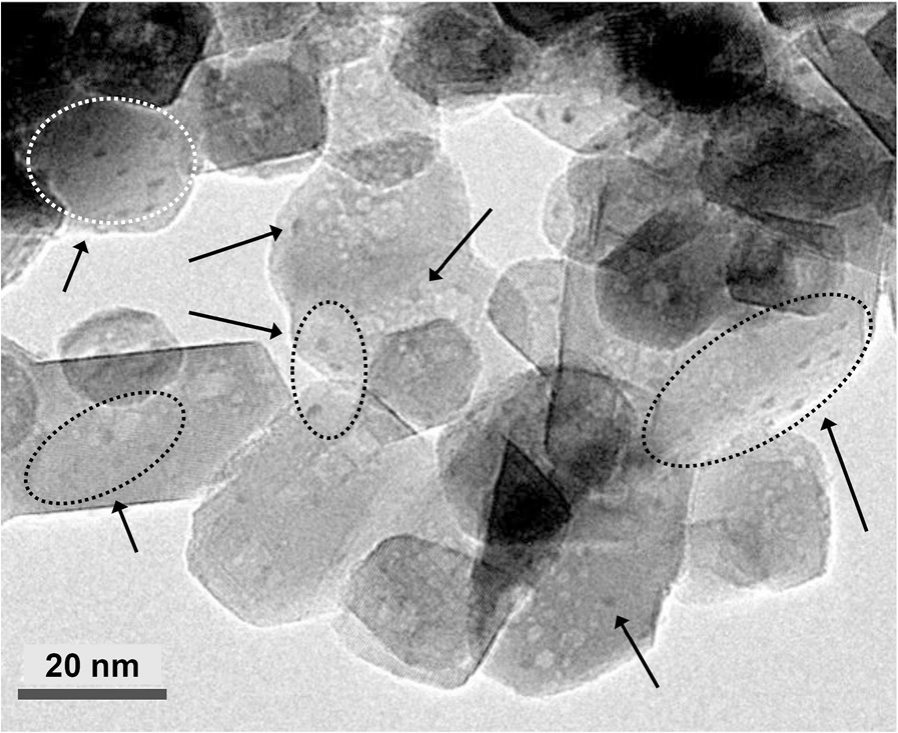
**TEM image of the (CH**_**3**_**CH**_**2**_**NH**_**3**_**)PbI**_**3**_-**deposited TiO**_**2**_**nanoparticles.** Arrows indicate (CH_3_CH_2_NH_3_)PbI_3_ nanodots.

Figure 2 shows TEM image of the (CH_3_CH_2_NH_3_)PbI_3_ deposited on TiO_2_ nanoparticles, where the (CH_3_CH_2_NH_3_)PbI_3_ dots are clearly seen and sparsely distributed on the TiO_2_ surface. This indicates that spin coating of the solution containing CH_3_CH_2_NH_3_I and PbI_2_ leads to (CH_3_CH_2_NH_3_)PbI_3_ dots on the TiO_2_ surface. The average size of the deposited (CH_3_CH_2_NH_3_)PbI_3_ is estimated to be about 1.8 nm in diameter.

**Figure 3:**
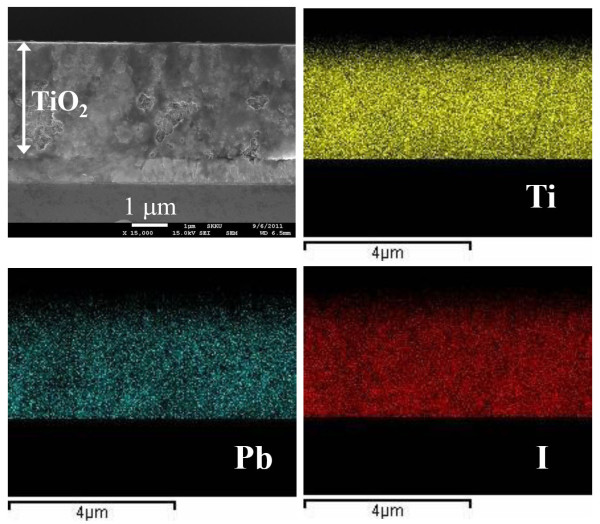
**Cross-sectional SEM micrographs of (CH**_**3**_**CH**_**2**_**NH**_**3**_**)PbI**_**3**_-**deposited TiO**_**2**_**film and EDS maps for titanium, lead, and iodine.**

Figure 3 shows cross-sectional EDS mapping, where Pb and I are well distributed three-dimensionally in the mesoporous TiO_2_ film. Atomic percentages from EDS elemental analysis are found to be 1.66% and 4.74% for Pb and I, respectively, which indicates that the ratio of Pb to I is close to 1:3.

**Figure 4:**
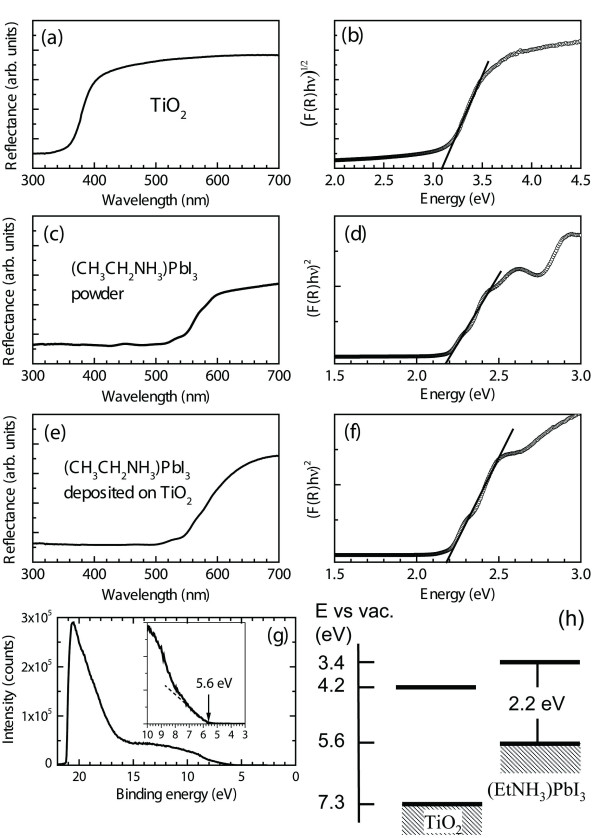
**Diffuse reflectance spectra, UPS spectrum, and schematic energy profile for (CH**_**3**_**CH**_**2**_**NH**_**3**_**)PbI**_**3**_**.** Diffuse reflectance spectra and the transformed Kubelka-Munk function for (**a**, **b**) the bare TiO_2_, (**c**, **d**) the powdered (CH_3_CH_2_NH_3_)PbI_3_, and (**e**, **f**) the (CH_3_CH_2_NH_3_)PbI_3_ deposited on TiO_2_. (**g**) UPS spectrum and (**h**) schematic energy profile for (CH_3_CH_2_NH_3_)PbI_3_. In UPS spectrum, binding energy was adjusted with respect to He-I (21.22 eV).

To determine optical bandgap and valence band position, UV–vis reflectance and UPS measurements are performed. Figure 4a,b,c,d,e,f shows the diffuse reflectance spectra and the transformed Kubelka-Munk spectra for the bare TiO_2_ nanoparticle, the powdered (CH_3_CH_2_NH_3_)PbI_3_, and the deposited (CH_3_CH_2_NH_3_)PbI_3_ on the TiO_2_ surface. The dependence of the optical absorption coefficient with the photon energy has been known to help to study the type of transition of electrons and semiconductors' bandgap energy as well [18]. The optical absorption coefficient (*α*) can be calculated using reflectance data according to the Kubelka-Munk equation [19], $\text{F}\left(R\right)=\alpha =\frac{{\left(1-R\right)}^{2}}{2R}$, where *R* is the reflectance data. The incident photon energy (hν) and the optical bandgap energy (*E*_g_) are related to the transformed Kubelka-Munk function, ${\left[\text{F}\left(R\right)\text{hν}\right]}^{\frac{1}{p}}=\text{A}\left(\text{hν}–E_{\text{g}}\right)$, where *A* is the constant depending on transition probability and *p* is the index that is related to the optical absorption process. Theoretically, *p* equals to 2 or ½ for an indirect or direct allowed transition, respectively. The *E*_g_ of the bare TiO_2_ determined based on indirect transition is 3.1 eV, which is well consistent with the data reported elsewhere [19]. For the case of (CH_3_CH_2_NH_3_)PbI_3_, a transformed Kubelka-Munk function can be constructed by plotting [F(*R*)]^2^ against the photon energy, which is indicative of direct transition. As shown in Figure 4c,d,e,f, an *E*_g_ of *ca.* 2.2 eV is estimated for both the powdered (CH_3_CH_2_NH_3_)PbI_3_ and the deposited one. According to UPS spectrum, the valence band energy (*E*_VB_) of (CH_3_CH_2_NH_3_)PbI_3_ is determined to be 5.6 eV with respect to vacuum level. Therefore, from the *E*_g_ and the *E*_VB_ values, conduction band energy (*E*_CB_) is estimated to be 3.4 eV, which is 0.8 eV higher than that of the *E*_CB_ for TiO_2_ (4.2 eV versus vacuum).

**Figure 5:**
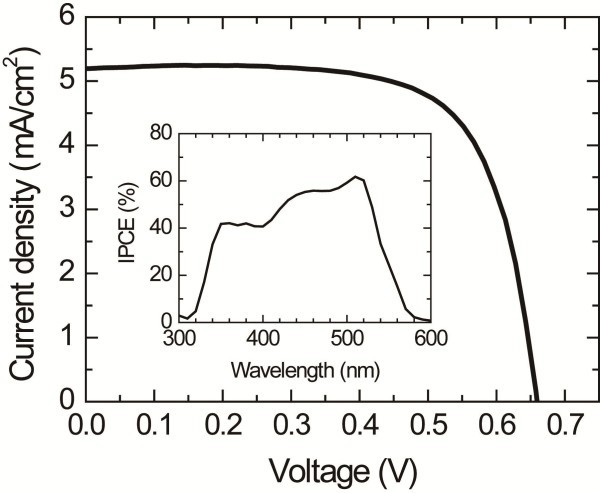
**Photocurrent density-voltage curve of the (CH**_**3**_**CH**_**2**_**NH**_**3**_**)PbI**_**3**_-**sensitized solar cell under AM 1.5 G one-sun light intensity.** The 2 H perovskite-type (CH_3_CH_2_NH_3_)PbI_3_-sensitized TiO_2_ layer was heated at 100°C for 15 min. The active area and TiO_2_ film thickness were 0.323 cm^2^ and 5.4 μm, respectively. The inset shows the IPCE spectrum.

Figure 5 shows the photovoltaic property of the (CH_3_CH_2_NH_3_)PbI_3_-sensitized solar cell, where I_3_^−^/I^−^ redox electrolyte is employed. A photocurrent density of 5.2 mA/cm^2^, a voltage of 0.660 V, and a fill factor of 0.704 are observed at AM 1.5 G one sun (100 mW/cm^2^) illumination, leading to an overall conversion efficiency of 2.4%. Incident photon-to-current conversion efficiency (IPCE) spectrum shows that the electron excitation starts to occur at around 570 nm, which is consistent with the estimated *E*_g_ of *ca.* 2.2 eV.

## Conclusions

We synthesized a new nanocrystalline sensitizer based on organic–inorganic hybridization. The crystal structure of the synthesized (CH_3_CH_2_NH_3_)PbI_3_ was determined to be 2 H perovskite-type orthorhombic phase. The optical bandgap was estimated to be *ca.* 2.2 eV, and the valence band energy position was determined to be 5.6 eV based on UPS measurement. The conduction band edge position of (CH_3_CH_2_NH_3_)PbI_3_ was 0.8 eV higher than that of TiO_2_, which allowed injection of photo-excited electrons from (CH_3_CH_2_NH_3_)PbI_3_ to TiO_2_. Under full sun illumination, the (CH_3_CH_2_NH_3_)PbI_3_-sensitized solar cell showed an overall conversion efficiency of 2.4%.

## Competing interests

The authors declare that they have no competing interests

## Authors' contributions

J-HI carried out the synthesis of perovskite materials and the fabrication of solar cell devices. JC and S-JK carried out the X-ray diffraction measurement and structure analysis. N-GP contributed to the conception and design of experiments, data interpretation, and writing of the manuscript. All authors read and approved the final manuscript.

## References

[B1] Alivisatos AP Perspectives on the physical chemistry of semiconductor nanocrystals J Phys Chem 1996 100 13226 10.1021/jp9535506

[B2] Alivisatos AP Semiconductor clusters, nanocrystals, and quantum dots Science 1996 271 933 10.1126/science.271.5251.933

[B3] Gaponenko Optical properties of semiconductor nanocrystals 1998 Cambridge University Press, Cambridge

[B4] Lee J Sundar VC Heine JR Bawendi MG Jensen KF Full color emission from II-VI semiconductor quantum dot-polymer composites Adv Mater 2000 12 1311

[B5] Michalet X Pinaud FF Bentolila LA Tsay JM Doose S Li JJ Sundaresan G Wu AM Gambhir SS Quantum dots for live cells, in vivo imaging, and diagnostics Science 2005 307 538 10.1126/science.1104274 15681376 PMC1201471

[B6] Nozik AJ Quantum dot solar cells Physica E 2002 14 115 10.1016/S1386-9477(02)00374-0

[B7] Hetsch F Xu XQ Wang HK Kershaw SV Rogach AL Semiconductor nanocrystal quantum dots as solar cell components and photosensitizers: material, charge transfer, and separation aspects of some device topologies J Phys Chem Lett 2011 2 1879 10.1021/jz200802j

[B8] Kamat PV Quantum dot solar cells. Semiconductor nanocrystals as light harvesters J Phys Chem C 2008 112 18737

[B9] Ruhle S Shalom M Zaban A Quantum-dot-sensitized solar cells Chemphyschem 2010 11 2290 10.1002/cphc.201000069 20632355

[B10] Im SH Lim CS Chang JA Lee YH Maiti N Kim HJ Nazeeruddin MdK Grätzel M Seok SI Toward interaction of sensitizer and functional moieties in hole-transporting materials for efficient semiconductor-sensitized solar cells Nano Lett 2011 11 4789 10.1021/nl2026184 21961842

[B11] Im JH Lee CR Lee JW Park SW Park NG 6.5% efficient perovskite quantum-dot-sensitized solar cell Nanoscale 2011 3 4088 10.1039/c1nr10867k 21897986

[B12] Pena MA Fierro JLG Chemical structures and performance of perovskite oxides Chem Rev 1981 2001 101 10.1021/cr980129f 11710238

[B13] Bhalla AS Guo RY Roy R The perovskite structure–a review of its role in ceramic science and technology Mater Res Innov 2000 4 3 10.1007/s100190000062

[B14] Kim MJ Lee CR Jeong WS Im JH Ryu TI Park NG Unusual enhancement of photocurrent by incorporation of Bronsted base thiourea into electrolyte of dye-sensitized solar cell J Phys Chem C 2010 114 19849 10.1021/jp107437h

[B15] Werner PE Eriksson L Westdahl M TREOR, a semi-exhaustive trial-and-error powder indexing program for all symmetries J Appl Cryst 1985 18 367 10.1107/S0021889885010512

[B16] Altamore A Cascarano G Giacovazzo C Guagliardi A Burla MC Polidori G Camalli M SIR92–a program for automatic solution of crystal structures by direct methods J Appl Cryst 1994 27 435

[B17] Rodriguez-Carvajal J FULLPROF 2000: A Rietveld Refinement and Pattern Matching Analysis Program 2008 Laboratoire Léon Brillouin (CEA-CNRS), France

[B18] Tauc J Grigorovici R Vancu A Optical properties and electronic structure of amorphous germanium Phys Stat Sol (b) 1966 15 627 10.1002/pssb.19660150224

[B19] Lin H Huang CP Li W Ni C Shah SI Tseng YH Size dependency of nanocrystalline TiO2 on its optical property and photocatalytic reactivity exemplified by 2-chlorophenol Appl Catal B-Environ 2006 68 1 10.1016/j.apcatb.2006.07.018

